# GP96 is over-expressed in oral cavity cancer and is a poor prognostic indicator for patients receiving radiotherapy

**DOI:** 10.1186/1748-717X-6-136

**Published:** 2011-10-12

**Authors:** Chien-Yu Lin, Ting-Yang Lin, Hung-Ming Wang, Shiang-Fu Huang, Kang-Hsing Fan, Chun-Ta Liao, I-How Chen, Li-Yu Lee, Yen-Liang Li, Yin-Ju Chen, Ann-Joy Cheng, Joseph T Chang

**Affiliations:** 1 Department of Radiation Oncology, Chang Gung Memorial Hospital, Taoyuan 333, Taiwan; 2 Department of Medical Biotechnology, Chang Gung University, Taoyuan 333, Taiwan; 3 Department of Medical Oncology, Chang Gung Memorial Hospital, Taoyuan 333, Taiwan; 4 Department of Otorhinolaryngology, Chang Gung Memorial Hospital, Taoyuan 333, Taiwan; 5 Department of Pathology, Chang Gung Memorial Hospital, Taoyuan 333, Taiwan; 6 Graduate Institute of Clinical Medical Science, Chang Gung University, Taoyuan 333, Taiwan; 7 Department of Medicine, Chang Gung University, Taoyuan 333, Taiwan

**Keywords:** GP96, oral cavity cancers, prognosis, radioresistance

## Abstract

**Background:**

Oral cavity cancers (ORC) are the most common cancers, and standard treatment is radical surgery with postoperative radiotherapy. However, locoregional failure remains a major problem, indicating radioresistance an important issue. Our previous work has shown that GP96 contributed to radioresistance in nasopharyngeal and oral cancer cell lines. In this study, we determined clinical significance of GP96 in ORC by evaluation of GP96 expression and its association with disease prognosis in patients receiving radiotherapy

**Methods:**

Total of 79 ORC patients (77 males, median age: 48 years old) receiving radical surgery and postoperative radiotherapy between Oct 1999 and Dec 2004 were enrolled. Patients in pathological stages II, III and IV were 16.5%, 16.5% and 67%, respectively. For each patient, a pair of carcinoma tissue and grossly adjacent normal mucosa was obtained. GP96-expression was examined by western blot analysis, and the association with clinicopathological status was determined.

**Results:**

Three-year locoregional control (LRC), distant metastasis-free survival (DMFS), disease-specific survival (DSS) and overall survival (OS) rates were 69%, 79%, 63% and 57%, respectively. We found that 55 patients (70%) displayed GP96-overexpression in the tumor tissue, which correlated with a higher pN stage (p = 0.020) and tumor depth (> 10 mm) (p = 0.045). Nodal extracapsular spreading (ECS) and GP96-overexpression predicted adverse LRC (p = 0.049 and p = 0.008). When stratified by nodal ECS, the adverse impact of GP96 remained significant in three-year LRC (p = 0.004). In multivariate analysis, GP96-overexpression was also an independent predictor of LRC, DSS and OS (p = 0.018, p = 0.011 and p = 0.012).

**Conclusion:**

GP96 may play roles in radioresistance which attributes to tumor invasiveness in oral cancer patients receiving radiotherapy. GP96 may serve as a novel prognostic marker of radiotherapy. However, further independent studies are required to validate our findings in a larger series.

## Background

Oral cavity cancers (ORC) are among the most common cancers in the world [1]. Epidemiological studies have shown strong associations between ORC and the use of tobacco, alcohol and betel quid [2]. The standard treatment for ORC is radical surgery [3]. Postoperative radiotherapy (Postop-RT) with/without concurrent chemotherapy is added to eliminate microscopic tumor cells in high-risk patients. However, locoregional failure remains a major problem if the tumor is radioresistant [4-7].

Heat shock protein (Hsp) is a highly conserved molecular chaperone protein that functions as biochemical regulators of cell growth, apoptosis, and homeostasis. It is up-regulated under stress conditions, such as starvation, hypoxia, heat, virus infection and neoplasia [8,9]. Hsp GP96, also known as glucose-regulated protein 94 (GRP94), is a member of the Hsp 90 family [10]. It plays an important role in regulating mitogenesis, cell cycle and apoptosis [8,9,11]. In addition, GP96 has been found to induce protective tumor-specific immunity [11]. Recently, aberrant GP96-expression has been observed in several cancers [12,13], suggesting a link between neoplasms and GP96-expression. Our previous work has shown that GP96 contributed to radioresistance in nasopharyngeal carcinoma (NPC) and ORC cell lines [14,15], indicating that this molecule may affect the efficacy of radiotherapy. In this study, we investigated the clinical significance of GP96 and the impact on treatment outcome in ORC patients with Postop-RT.

## Materials and methods

### Patients and specimens

We obtained tissue bank specimens from ORC patients visiting the Chang Gung Memorial Hospital-Linko between Oct 1999 and Dec 2004. Samples were from 79 patients with newly diagnosed non-metastatic ORC receiving radical surgery followed by Postop-RT. A grossly normal sample of oral mucosal tissue as well as a tumor specimen was collected. This study was approved by the Institutional Review Broad of the Human Investigation Committee in our institution.

### Staging and Treatments

The pre-treatment workup included a chest X-ray, liver ultrasound and bone scan to exclude distant metastases. F18-FDG PET (18-fluoro, 2-fluoro-2-deoxy-D-glucose, positron emission tomography) was incorporated after Oct. 2003. Computed tomography (CT) or magnetic resonance imaging (MRI) was used to determine tumor burden. Radical surgery was defined as a wide excision with a 1-2 cm safety margin with/without immediate free-flap reconstruction. Mandibulectomy or maxillectomy were performed as dictated by tumor extension or margin space. Ipsilateral elective neck dissection was used for clinical N0 patients and radical neck dissection for clinical N+ patients. Intraoperative frozen examinations were performed to ensure adequate margins. The definition of an adequate margin was a tumor-free margin of at least 5 mm according to final pathological report. All tumor stage evaluations were revised according to the 2002 AJCC pathological staging criteria.

Postop-RT was scheduled within 4-8 weeks of surgery and was administered as 6 megavolt x-ray generated by a linear accelerator. Conventional radiotherapy techniques, 2-dimensional planning or 3-dimensional conformal radiotherapy were used in early patients, while intensity-modulated radiotherapy (IMRT) was incorporated after 2001. Conventional techniques consisted of bilateral-opposing and lower-anterior neck portals. Neck boosts by megavolt electron were used for sparing spinal cord after 46 Gy. Doses of 1.8-2 Gy/fraction were given in 5 fractions per week. Initial prophylactic doses of 46-50 Gy were for all risk areas and a further boost of 60 Gy for the primary tumor bed and involved nodal areas. Elevated doses of 66 Gy in combination with concurrent chemotherapy were used in patients with positive margins, nodal extracapsular spreading (ECS) or pathological multiple nodal metastasis. Concurrent chemotherapy was administered with intravenous Cisplatin 50 mg/m2 and oral 5-FU analogue 1400 mg/m2 combined with leucovorin 60 mg on a biweekly schedule during radiotherapy. Patients were closely followed for at least three years or until death. Patient status as of the last follow-up was recorded at the last outpatient visit, telephone interview or date of death.

### Tissue processing, protein extraction and western blot analysis

For each tissue, cellular proteins were extracted and the level of GP96 protein was determined by western blot method, similarly as previously described.^15 ^Briefly, total of 20 μg tissues protein were separated by 8% SDS-polyacrylamide gel electrophoresis and transferred to a nitrocellulose membrane. The membrane was hybridized with an anti-GP96 antibody (NeoMarkers, Fremont, CA, USA) and subsequently incubated with secondary antibodies conjugated to horseradish peroxidase. The blots were developed using Renaissance chemiluminescence reagent (NEN Life Science Products, MA, USA) following autoradiography. To determine the relative expression of GP96 in tumor tissue, the band density of each tumor sample was compared with the normal oral mucosa sample taken from the same patient after normalization to an internal control (actin). GP96-overexpression was defined as a 1.5-fold increase in lesion tissue as compared with the normal oral mucosa. The level of GP96-expression and its associations with clinicopathological parameters and treatment outcomes were analyzed.

### Statistical analysis

Time intervals were calculated from the end of RT to the events of interest. Locoregional control (LRC) was defined as freedom from relapse at the primary site or neck, distant relapse for distant metastasis-free survival (DMFS), and either one for disease-free survival (DFS). Disease-specific survival (DSS) was defined as survival until death from the disease or treatment-related toxicities, and any other cause for overall survival (OS). Relapse events were defined by imaging findings, clinical or pathological examination. Commercial statistical software (SPSS 13.0; SPSS, Chicago, IL) was used for data analysis. Variables that might affect outcomes were evaluated using the chi-squared test, independent t-test or Fisher's exact test as appropriate. Survival curves were calculated by the Kaplan-Meier method with a log-rank test for univariate analysis. A stepwise Cox-regression model for multivariate analysis was used for further analysis of potentially significant variables.

## Results

### Patient characteristics and treatment outcome

Our study included 79 patients, and median age was 48 years old (range 30-75). The patient characteristics were listed in Table 1. Pre-treatment imaging was performed using MRI (n = 42, 53%), CT scan (n = 32, 41%) or both (n = 5, 6%). Thirteen (16.5%) patients also had F18-FDG PET scans to aid diagnosis. Primary tumor site were buccal (n = 57, 72%) and majority of patients were stage IV disease (67%). The detailed T-N distribution is shown in Table 2.

**Table 1:** Association between GP96 overexpression and clinicopathological parameters

	Total patients		GP96 over-expression				
				
			No		Yes		
		
	**No**.	%	**No**.	%	**No**.	%	*P*
Gender							0.518*
Male	77	97.5	23	30	54	70	
Female	2	2.5	1	50	1	50	
Age (years)							0.573
≤ 48	44	51	11	27.5	29	72.5	
> 48	39	49	13	33	26	67	
Smoking							0.573*
No	14	18	4	29	10	71	
Yes	65	82	20	31	45	69	
Drinking							0.237
No	19	24	4	21	15	79	
Yes	60	76	20	33	40	67	
Betel-quid chewing							0.427*
No	14	18	5	36	9	64	
Yes	65	82	19	29	46	71	
Stage							0.349*
I-II	13	16.5	5	38.5	8	61.5	
III-IV	66	83.5	19	29	47	71	
T stage							0.390
T1-2	36	46	12	33	24	67	
T3-4	43	54	12	28	31	72	
Nodal stage							0.020
N0-1	44	56	18	41	26	59	
N2	35	44	6	17	29	83	
Nodal ECS							0.183
No	45	57	16	33	29	67	
Yes	34	43	8	23.5	26	76.5	
Differentiation							0.654
Well	24	30	9	37.5	15	62.5	
Moderate	47	60	13	28	34	72	
Poor	8	10	2	25	6	75	
Depth (millimeters)							0.045
≤10	30	38	13	43	17	57	
> 10	49	62	11	22	38	78	
Skin invasion							0.641*
No	72	91	22	31	50	69	
Yes	7	9	2	29	5	71	
Bone invasion							0.402
No	56	71	18	32	38	68	
Yes	23	29	6	26	17	74	
Perineural invasion							0.424
No	53	67	15	28	38	72	
Yes	24	30	8	33	16	67	
Missing	2	3					
Blood Vessel invasion							
No	72	91	22	31	50	69	0.528*
Yes	5	6	1	20	4	80	
Missing	2	3					
Lymphatic vessel invasion							0.069*
No	65	82	22	34	43	66	
Yes	12	15	1	8	11	92	
Missing	2	3					
Margin status (millimeters)							0.605*
≥ 5	66	83.5	20	30	46	70	
< 5	13	16.5	4	31	9	69	

* Fisher's exact test

*Abbreviations: *ECS = extracapsular spreading

**Table 2:** Distribution of pathological T and N stage

	Pathological N stage			
		
Pathological T stage	N0	N1	N2	Total
T1	0	2	2	4 (5)
T2	13	6	13	32 (40.5)
T3	3	2	8	13 (16.5)
T4	12	6	12	30 (38)
Total	28 (36)	16 (20)	35 (44)	79 (100)

Values in parentheses are percentages

Radical surgery followed by Postop-RT was performed in this cohort. The median nearest resection margin was 8 mm (range: 1-15), and no patients had positive margins. Neck dissection was ipsilateral (n = 67, 85%), bilateral (n = 10, 13%), and none (n = 1). For Postop-RT, elective neck irradiation was ipsilateral (n = 58, 73%) and bilateral in remaining cases. The median time interval between operation and radiotherapy was 5.5 weeks (range: 2.7-15), and a median dose of 66 Gy (range: 56-68) in median of 7 weeks (rang: 5.8-16). Thirty-eight (48%) patients received concurrent chemotherapy.

**Figure 1:**
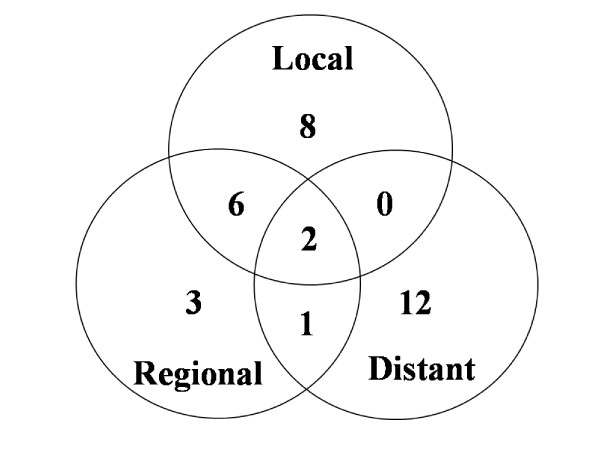
**Treatment failure pattern**.

The median follow-up time was 4.3 years (range, 2.1-8.0). At the end of the study, 39 patients remained alive and 40 had died. Deaths were due to locoregional disease (n = 14), distant metastasis (n = 14), second primary malignancy (n = 3), intercurrent disease (n = 8) and traffic accident (n = 1). Thirty-two patients suffered from recurrent disease and the detailed failure pattern is shown in Figure 1. A total of 20 patients (25%) had locoregional recurrence and 15 patients (19%) had distant metastasis. All locoregional failures were infield recurrences except for two outfield neck recurrences.

### GP96 is over-expressed in oral cavity cancers and predicts poorer treatment outcome

**Figure 2:**
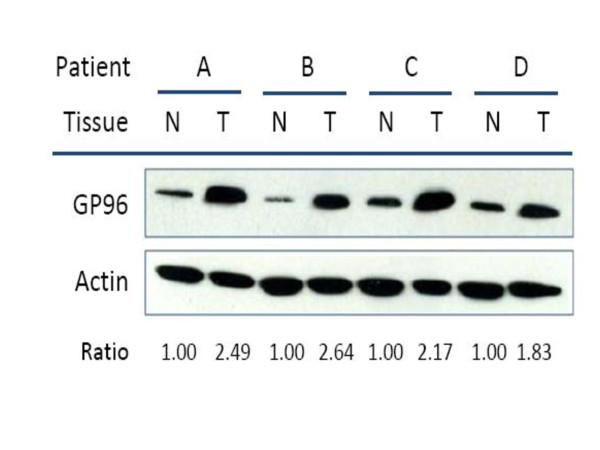
**GP96 is highly expressed in advanced oral cancer tissues**. Four pairs of normal (N) and tumor (T) tissues from oral cancer patients were examined. Protein expression was determined by western blot analysis. Actin protein expression served as an internal control.

Using the criterion of a 1.5-fold differential expression in tumor tissue compared to the normal counterpart, there were 9 out of 79 patients (11.4%) whose tumor had GP96 under-expression (≤ 1.5-fold), 11 patients (19.0%) equal expression (between 0.67- to 1.5-fold), and 55 patients (69.6%) over-expression (≥ 1.5-fold, Figure 2). However, the treatment outcomes had no statistical difference between under- and equal-expressed patients. Therefore, we categorized GP96 under- and equal-expression into the same group for analysis. The associations between GP96 over-expression and clinicopathological factors are summarized in Table 1. Significant correlations were found between GP96-overexpression and nodal stage *(p = 0.020*) or tumor depth (*p = 0.045*). Other associations were not observed.

**Figure 3:**
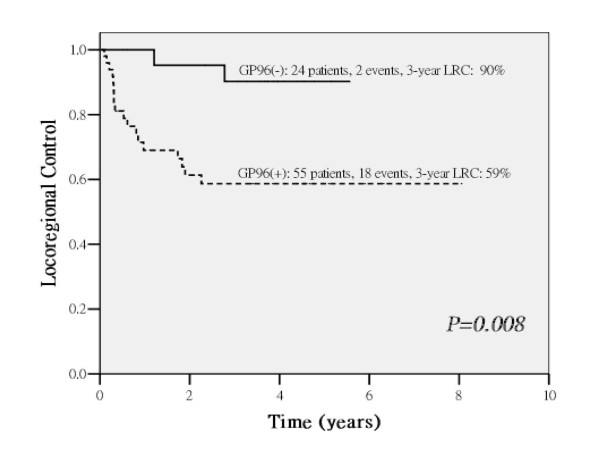
**GP96-overexpression is a poorer prognostic factor on locoregional control (LRC), as shown by the survival curves calculated by the Kaplan-Meier method**. GP96(+): GP96 overexpression, as the protein level is 1.5-fold higher in the tumor tissue compared to the normal counterpart; GP96(-): GP96 non-overexpression, as the protein level is comparable or lower (≤ 1.5-fold) compared to the normal counterpart.

The three-year overall LRC, DMFS, DFS, DSS and OS were 69%, 78.6%, 57.5%, 63.3% and 56.6%, respectively. The treatment prognosis evaluation by univariate analysis and multivariate analysis were summarized in Table 3 and Table 4. In univariate analysis, patients with the status of GP96-overexpression in tumors predicts poorer treatment outcome on 3-year LRC (*p = 0.008*, Figure 3), DMFS (*p = 0.018*), DFS (*p = 0.001*), DSS (*p = 0.001*) and OS (*p = 0.003*). In multivariate analysis, GP96-overexpression remained independent significance on LRC *(p = 0.018)*, DFS *(p = 0.006)*, DSS *(p = 0.011)*, OS *(p = 0.012)*, and marginal effect on DMFS *(p = 0.072)*.

**Table 3:** Univariate analysis of 3-year survival

		LRC (%)	*p*	DMFS (%)	*p*	DFS (%)	*p*	DSS (%)	*p*	OS (%)	*p*
Stage	I-II III-IV	81.8 66.4	0.245	84.6 77.5	0.548	69.2 55.1	0.282	76.9 60.4	0.208	76.9 54.2	0.033
T stage	T1-2 T3-4	77.5 61.6	0.145	81.9 75.6	0.458	66.3 49.6	0.139	71.3 55.9	0.144	66.7 50.4	0.021
N stage	N0-1 N2-3	74.1 62.2	0.196	87.5 66.8	0.023	66.7 45.8	0.024	75.8 46.6	0.007	72.3 40.0	0.002
Depth (millimeters)	≤10 > 10	61.2 74.8	0.367	90.0 71.7	0.118	55.1 58.8	0.938	67.9 60.3	0.694	62.4 55.1	0.417
Differentiation	Well Moderate Poor	72.0 67.1 75.0	0.818	86.6 82.5 37.5	0.003	66.0 56.6 37.5	0.264	70.2 64.1 37.5	0.203	66.7 56.8 37.5	0.580
Nodal ECS	No Yes	78.1 53.8	0.049	87.9 63.7	0.019	70.7 38.3	0.005	79.5 39.4	0.001	75.6 34.3	0.001
Skin invasion	No Yes	71.6 41.7	0.153	81.3 51.4	0.076	60.5 28.6	0.071	67.0 28.6	0.033	60.8 28.6	0.204
Bone invasion	No Yes	71.6 62.7	0.407	74.5 90.7	0.226	57.7 56.9	0.871	64.2 60.2	0.940	58.9 54.8	0.251
Perineural invasion	No Yes	68.1 71.9	0.717	81.3 69.2	0.295	58.6 51.9	0.349	67.8 50.0	0.170	62.0 45.8	0.318
Blood vessel invasion	No Yes	67.5 80.0	0.729	78.0 75.0	0.999	56.0 60.0	0.891	62.4 60.0	0.909	56.6 60.0	0.741
Lymphatic vessel invasion	No Yes	67.6 75.8	0.845	81.9 55.6	0.014	58.1 46.3	0.218	65.1 45.8	0.106	59.6 41.7	0.117
Margin status (millimeters)	≥ 5 < 5	67.1 80.8	0.406	77.3 84.6	0.737	55.4 68.4	0.517	59.2 83.3	0.152	54.1 65.9	0.157
Concurrent chemotherapy	No Yes	70.8 67.8	0.613	89.3 66.0	0.014	65.2 48.9	0.071	72.6 52.4	0.506	70.6 44.3	0.022
GP96 overexpression	No Yes	90.2 58.7	0.008	95.8 69.5	0.018	86.5 44.7	0.001	95.7 49.0	0.001	87.5 44.9	0.003

*Abbreviation: *LRC = locoregional control; DMFS = distant metastasis-free survival; DFS = disease-free survival; DSS = disease-specific survival; OS = overall-survival; ECS = extra-capsular spreading.

**Table 4:** Multivariate analysis of significant risk factors for survival

	P	Hazard	% 95 CI
**LRC**			
GP96 over-expression	0.018	5.808	1.345-25.093
**DMFS**			
GP96 over-expression	0.072	6.504	0.848-49.871
Skin invasion	0.020	4.973	1.289-19.190
Differentiation	0.015	3.975	1.309-12.065
Concurrent chemotherapy	0.052	3.276	0.992-10.821
**DFS**			
GP96 over-expression	0.006	5.326	1.614-17.580
Nodal ECS	0.019	2.337	1.148-4.758
**DSS**			
GP96 over-expression	0.011	6.532	1.530-27.890
Nodal ECS	0.014	2.698	1.225-5.942
Skin invasion	0.040	2.806	1.046-7.526
**OS**			
GP96 over-expression	0.012	3.355	1.302-8.648
Nodal ECS	0.005	2.533	1.332-4.819

*Abbreviation: *LRC = locoregional control; DMFS = distant metastasis-free survival; DFS = disease-free survival; DSS = disease-specific survival; OS = overall-survival; ECS = extra-capsular spreading.

### Contribution of GP96-expression to LRC in N stage or ECS-stratified populations

**Figure 4:**
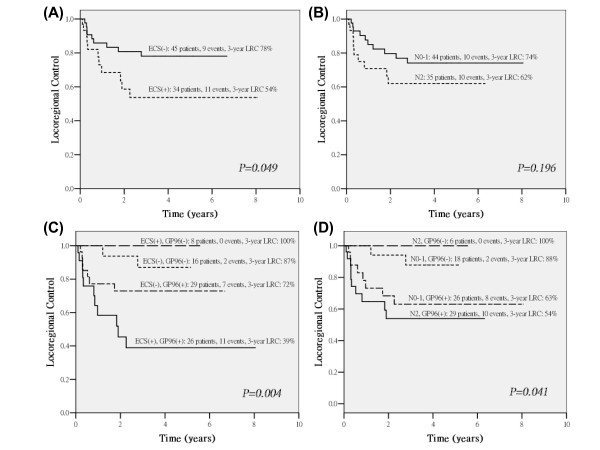
**Contribution of GP96-expression on locoregional control (LRC) in nodal ECS (A) or pN2 (B) stage-stratified patients, and provides additional information when stratified by risk of ECS (C) or N stage (D)**. All the survival curves were calculated by the Kaplan-Meier method. ECS(+): positive for nodal ECS; ECS(-): negative for nodal ECS; N0-1: N0-1 stage; N2: N2 stage; GP96(+): GP96 overexpression, as the protein level is 1.5-fold higher in the tumor tissue compared to the normal counterpart; GP96(-): GP96 non-overexpression, as the protein level is comparable or lower (≤ 1.5-fold) 1.5-fold) compared to the normal counterpart.

According to well-known pathological risk factors, patients displaying positive margins, nodal ECS or pN2 stage disease are at high risk in terms of selection for concurrent chemoradiotherapy [5-7,16]. Therefore, we further examined the effect of GP96-expression in relation to these indicators. Because no case with positive margins was available in this study, only pN2 stage disease and nodal ECS were used. In this study, 3-year LRC for nodal ECS or pN2 were 54% and 62%, respectively (Figure 4A-B). Further stratified by GP96-expression status, distinguishable four groups were observed. In patients with nodal ECS, GP96-overexpression still predicts poorer 3-year LRC 39% (*vs*. 100%) (Figure 4C) and also noted in patients with pN2 stage (54% *vs*. 100%) (Figure 4D). These results suggest that GP96 overexpression in tumor cells may be a significant predictor of poor prognosis for those patients receiving radiotherapy.

## Discussion

In this study, the treatment outcomes of ORC were comparable to previously published data [5-7]. Our data indicate that nodal ECS is a predictor of treatment outcomes by univariate analysis (Table 3). This effect was lost in the multivariate analysis of LRC, but it remained significant for other outcome (Table 4). The impact on treatment outcome of pN2 has only marginal effect. This observation agrees with our retrospective data indicating that concurrent chemoradiotherapy can overcome this negative effect, and considering of GP96-expression is another important issue.

GP96, a 94-100 kDa Ca^2+^-binding protein, is the most abundant protein in the endoplasmic reticulum (ER). It functions as a chaperone in ER, regulates mitogenesis, apoptosis, and antigenic-presenting immune response [8-11]. Up-regulation of GP96-expression has been reported under stress conditions, including starvation, hypoxia, heat, viral infection and neoplasia [8,9]. In the presence of stress, the final fate of cells may depend on their ability to resist stress. GP96 regulates cell fate by maintaining the intracellular Ca^2+ ^balance between the cytosol, ER and mitochondria. In this study, we examined GP96-expression in advanced ORC patients and found that GP96 is overexpressed in 70% of patients, which is consistent with previous findings indicating that GP96 is overexpressed in several human neoplasms [12,13,17]. It indicates that GP96 plays an important role in cancer development and continuous expression required for regulation and stabilizing tumor growth [13]. We also found that GP96-expression was correlated with tumor depth and N stage (Table 1). It is consistent with reports suggesting associations between elevated GP96-expression and tumor advanced stage or invasive ability. In addition, we found that GP96-overexpression was a strong independent prognostic factor for LRC, DFS, DSS and OS, although a marginal effect on DMFS (Table 3). It is consistent with a previous report indicating that GP96-expression serves as a poor prognostic factor in gastric carcinoma [13].

As mentioned above, GP96-expression was a strong predictor of LRC (*p = 0.008*, Table 3, Figure 3) and was the only independent predictor in the multivariate models (Table 4). We therefore hypothesize that GP96-expression is strongly associated with tumor radioresistance. Based on these observations, we further analyzed the effect of GP96-expression using stratification by well-established pathological risk factors such as nodal ECS or pN2 stage; the adverse effect of GP96-expression was still distinguishable (Figure 4C-D). We observed GP96-overexpression makes it poorer on LRC in patients with nodal ECS or pN2 stage, suggesting that GP96 may exhibit tumor radioresistance. Interestingly, in non-GP96-overexpression group, if patients with nodal ECS or pN2, which were high-risk patients selected for concurrent chemotherapy, it has inversely better 3-year LRC than low-risk patients who received Postop-RT alone (Figure 4C-D). This might be due to concurrent chemotherapy may effectively enhance tumor cell-killing in normal GP96-expression group.

In this study, our observations were consistent with previous reports that GP96-overexpression reduced radiosensitivity in cervical cancer and NPC cell lines [14,18]. In addition, it supports the clinical evidence comparable with our previous work on ORC cell lines: increasing of GP96-expression was observed in radioresistant sublines, and GP96-knockdown enhanced radiosensitivity via increasing G2/M arrest and reactive oxygen species levels [15]. Therefore, GP96 may play roles in radioresistance which attributes to tumor invasiveness in oral cancer patients receiving radiotherapy. GP96 may serve as a novel prognostic marker of radiotherapy. However, further independent studies are required to validate our findings in a larger series.

## List of abbreviations

(ORC): Oral cavity cancer; (Postop-RT): Postoperative radiotherapy; (Hsp): Heat shock protein; (NPC): Nasopharyngeal carcinoma; (CT): Computed tomography; (MRI): Magnetic resonance image; (IMRT): Intensity-modulated radiotherapy; (ECS): Extracapuslar spreading; (LRC): Locoregional control; (DSS): Disease-specific survival; (DMFS): Distant metastasis-free survival; (DFS): disease-free survival; (OS): Overall survival; (ER): Endoplasmic reticulum

## Competing interests

The authors declare that they have no competing interests.

## Authors' contributions

CYL, AJC and JTC prepared the study concept and design. CYL did the major manuscript writing. AJC and JTC did the major revision of the manuscript. CYL, YLL and TYL made the major contribution for tissue processing. CYL and AJC did the laboratory interpretation. CYL did the data analysis. HMW, SFH, KHF and JTC participated in the clinical data interpretation. CYL, HMW, SFH, KHF, CTL and IHC treated the patients and did the data collection. All authors read and approved the final manuscript
